# Christmas Number of the War Hospital Gazettes

**Published:** 1916-12-16

**Authors:** 


### Christmas Number of the War Hospital Gazettes.

How is it that the *Craigleith Hospital Chronicle* continues to be so full of mattter and so attractive in form, while, for instance, the *Gazette* of the 1st Eastern General Hospital, Cambridge, continues to complain of a dearth of talent, and seems to have to consider economy so carefully in the paper and style of printing which it employs? It is true that the Christmas number of the Cambridge Hospital is not before us as we write, but when we turn to the *Chronicle* of Craigleith Hospital the high standard achieved in matter and form is very striking. A coloured cover, thick paper, some sixty pages of articles and illustrations, make a brave show, and this generous treatment appears to be justified by a considerable number of advertisements. Apart from the black-and-white drawings, of which there are several, the articles cover a wide range. There is one on "The Dog in War, Past and Present," by Eve Blantyre Simpson; another of the series on "The Scottish Regiments," on the Gordon Highlanders, by J. A. MacDougall, M.D.; several more or less ambitious verses, and an account of the manner in which the second anniversary of the declaration of war was commemorated at Ajmer, India. This list of the more important contributions does not exhaust the contents, and among the illustrations the exuberant drawing of a Tank, entitled "A German Nightmare," deserves special mention. By very different means the Christmas number of the *Gazette* of the 3rd London General Hospital, Wandsworth, achieves similar success. Printed on small pages, with double columns and tiny type, it is made attractive with inset drawings which render each page decorative and interesting. It is, by comparison with the methods of the *Craigleith Chronicle*, the intensive method. The former C.O. sets a good example to other transferred members of the staff by contributing an article on his present surroundings, which happen to be Mesopotamia. Drawings by Corporal Tom Roberts, Lance-Corporal J. Hobson Lobley's "Night Scene in the Main Hall," the various silhouettes by Marjory Collins, are all worthy of mention. The photographs include portraits of Corporal Vernon Lorimer, an Australian patient, many of whose drawings have appeared, and of Lieutenant F. Derwent Wood, the sculptor, whose work as an artist and in what is called "oral surgery" is now well known.

